# Near-Complete Genome Sequence of a Newly Emerging Subgenotype of Atypical Porcine Pestivirus

**DOI:** 10.1128/MRA.00115-20

**Published:** 2020-04-02

**Authors:** Xiaoru Wang, Yongsheng Xie, Dongsheng He, He Yan

**Affiliations:** a School of Food Science and Engineering, South China University of Technology, Guangzhou, Guangdong Province, China; b College of Veterinary Medicine, South China Agricultural University, Guangzhou, Guangdong Province, China; Portland State University

## Abstract

This study reports the near-complete genome sequence of GD-CT4, a strain of a newly emerging subgenotype of atypical porcine pestivirus detected in a newborn piglet with congenital tremors in Guangdong Province, China. This sequence will improve the understanding of the epidemiology and genetic characteristics of atypical porcine pestivirus.

## ANNOUNCEMENT

Atypical porcine pestivirus (APPV), a newly discovered member of the genus *Pestivirus* within the family *Flaviviridae*, was first identified in the United States in 2015 through next-generation sequencing of swine serum samples (1). Subsequently, several reports described APPV as a prominent cause of congenital tremor (CT) type A-II in newborn piglets (2–5).

The GD-CT4 strain was derived from a newborn piglet with CT in October 2018 in Guangdong Province, China. The piglet’s brain, lung, heart, kidney, spleen, and lymph nodes were collected, homogenized using TissueLyser II (Qiagen, Hilden, Germany), diluted 10-fold with 0.1 M phosphate-buffered saline (pH 7.4), frozen and thawed thrice, and centrifuged at 8,000 × *g* for 30 min at 4°C. Viral RNA was extracted from supernatants using the QIAamp viral RNA minikit (Qiagen). Reverse transcription-PCR amplification for the APPV genome sequence was performed using 10 pairs of specific primers (6). The PCR products were purified and cloned into the pMD19-T vector (TaKaRa, Dalian, China) and sequenced in both directions by Sanger sequencing. Sequences were assembled and edited using DNAStar Lasergene 7.1. Fifty-eight previously sequenced APPV genomes and five non-APPV *Pestivirus* strain genomes were used for multiple sequence alignment analysis using MegAlign software (DNAStar v7.1, Madison, WI, USA) with the ClustalW method. Phylogenetic analysis was performed based on complete polyprotein sequences by the neighbor-joining (NJ) method with 1,000 bootstrap replicates in MEGA v.7.0.26 software.

**FIG 1:**
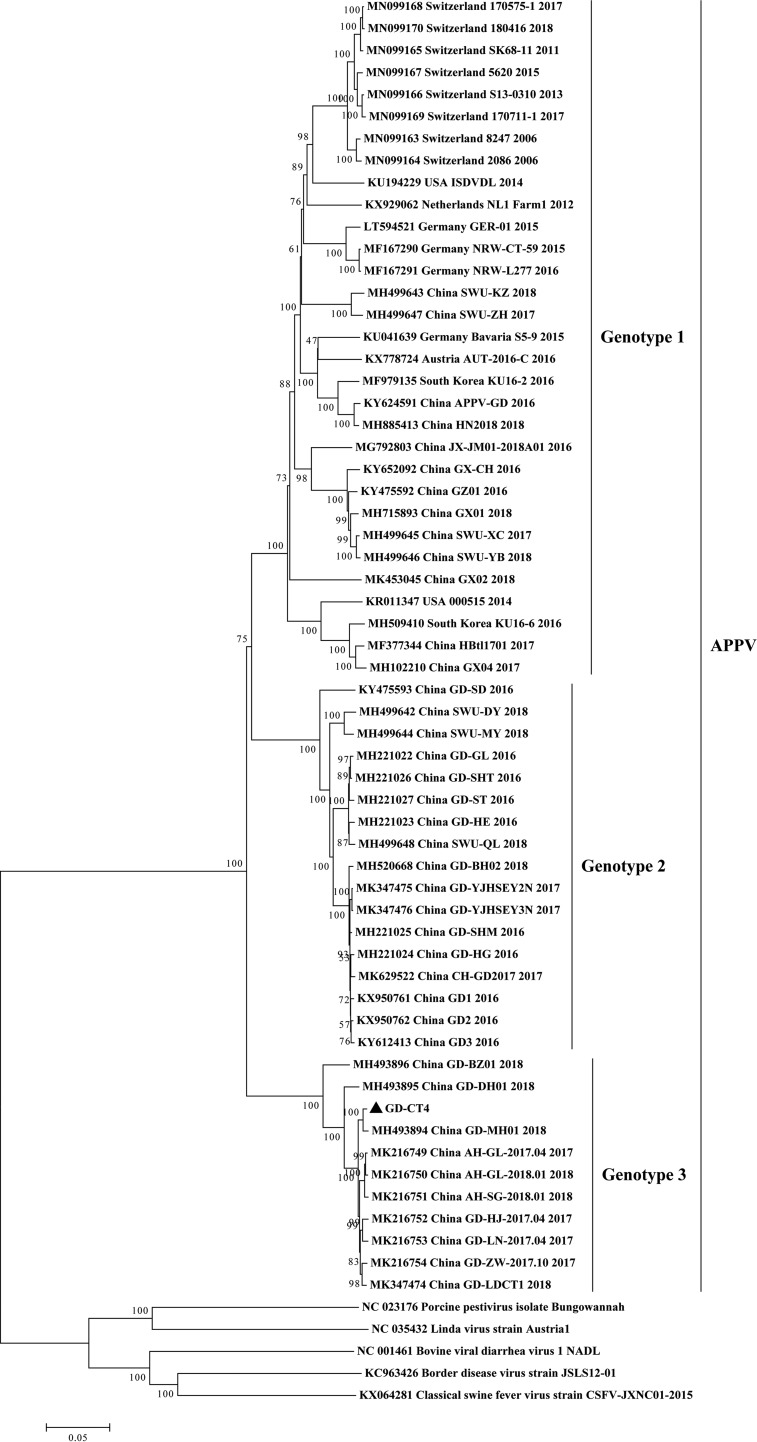
Phylogenetic reconstruction based on polyprotein sequences of pestivirus species. The phylogenetic tree was constructed by the neighbor-joining method using MEGA software (v.7.0.26) with 1,000 bootstrap replicates. The tree was drawn to scale, with branch lengths in the same units as those of the evolutionary distances used to infer the phylogenetic tree. The complete polyprotein sequence of APPV obtained in this study is marked with a black triangle.

APPV is known as a highly variable single-stranded RNA virus with an ∼11- to 12-kb genome that contains a long open reading frame (ORF) encoding a polyprotein of four structural proteins (C, Erns, E1, and E2) and eight nonstructural proteins (Npro, P7, NS2, NS3, NS4A, NS4B, NS5A, and NS5B) (1). The GD-CT4 sequence was 11,101 bp long, and its G+C content was 46.0%, obtaining 96.5% of the genome relative to the reference sequence (GenBank accession number MK216752). The missing nucleotides were located in noncoding regions at the genome ends. A complete ORF (10,908 bp) encoding a polyprotein of 3,635 amino acids was found between nucleotide positions 45 and 10952. Phylogenetic and homology analyses revealed that APPV could be classified into three genotypes, namely, 1, 2, and 3 (Fig. 1). The GD-CT4 strain shared nucleotide identities (80.9% to 82.3%, 80.8% to 81.2%, and 94.1% to 99.3%) and amino acid identities (90.8% to 91.8%, 90.5% to 91.3%, and 97.8% to 99.5%) with strains from genotypes 1, 2, and 3, respectively. GD-CT4 and 10 other strains from China formed genotype 3, a newly emerging subgenotype (6–8). Within the genotype 3 cluster, GD-CT4 displayed the closest relationship to strain GD-MH01-2018 (GenBank accession number MH493894) from Guangdong Province, China, by sharing 40 synonymous and 14 nonsynonymous ORF mutations. Notably, nonsynonymous substitutions occurred in the important functional genes NS2, NS3, NS4B, NS5A, and NS5B.

Although APPV genotype 3 has been detected in newborn piglets showing clinical signs of CT, within only three provinces in China (Guangdong, Jiangxi, and Anhui), it has caused great socioeconomic losses (6–8). Therefore, genomic information on this emerging subgenotype is invaluable for understanding the origin, evolution, and transmission pattern of APPV genotype 3.

### Data availability.

The GD-CT4 sequence is available in GenBank under accession number MN584737.
